# Procalcitonin-guided therapy in intensive care unit patients with severe sepsis and septic shock – a systematic review and meta-analysis

**DOI:** 10.1186/cc13157

**Published:** 2013-12-11

**Authors:** Anna Prkno, Christina Wacker, Frank M Brunkhorst, Peter Schlattmann

**Affiliations:** 1 Department of Medical Statistics, Computer Sciences and Documentation, Jena University Hospital, Bachstrasse 18, D-07743 Jena, Germany; 2 Center for Sepsis Control and Care (CSCC), Jena University Hospital, Jena, Germany; 3 Paul-Martini-Clinical Sepsis Research Unit, Center of Clinical Studies, Department of Anaesthesiology and Intensive Care Medicine, Jena University Hospital, Jena, Germany

## Abstract

**Introduction:**

Procalcitonin (PCT) algorithms for antibiotic treatment decisions have been studied in adult patients from primary care, emergency department, and intensive care unit (ICU) settings, suggesting that procalcitonin-guided therapy may reduce antibiotic exposure without increasing the mortality rate. However, information on the efficacy and safety of this approach in the most vulnerable population of critically ill patients with severe sepsis and septic shock is missing.

**Method:**

Two reviewers independently performed a systematic search in PubMed, Embase, ISI Web of Knowledge, BioMed Central, ScienceDirect, Cochrane Central Register of Controlled Trials, http://www.ClinicalTrials.gov and http://www.ISRCTN.org.

Eligible studies had to be randomized controlled clinical trials or cohort studies which compare procalcitonin-guided therapy with standard care in severe sepsis patients and report at least one of the following outcomes: hospital mortality, 28-day mortality, duration of antimicrobial therapy, length of stay in the intensive care unit or length of hospital stay. Disagreements about inclusion of studies and judgment of bias were solved by consensus.

**Results:**

Finally seven studies comprising a total of 1,075 patients with severe sepsis or septic shock were included in the meta-analysis.

Both hospital mortality (RR [relative risk]: 0.91, 95%CI [confidence interval]: 0.61; 1.36) and 28-day mortality (RR: 1.02, 95%CI: 0.85; 1.23) were not different between procalcitonin-guided therapy and standard treatment groups.

Duration of antimicrobial therapy was significantly reduced in favor of procalcitonin-guided therapy (HR [hazard ratio]: 1.27, 95%CI: 1.01; 1.53). Combined estimates of the length of stay in the ICU and in hospital did not differ between groups.

**Conclusion:**

Procalcitonin-guided therapy is a helpful approach to guide antibiotic therapy and surgical interventions without a beneficial effect on mortality. The major benefit of PCT-guided therapy consists of a shorter duration of antibiotic treatment compared to standard care.

Trials are needed to investigate the effect of PCT-guided therapy on mortality, length of ICU and in-hospital stay in severe sepsis patients.

## Introduction

Severe sepsis and septic shock are common diseases in ICUs, with high mortality rates [1,2]. According to more recent data from the Statistical Federal Office in Germany, there were 88,000 patients with severe sepsis or septic shock in 2011 in German hospitals, with associated hospital mortality rates of 43% for severe sepsis and 60% for septic shock, respectively [3]. To overcome this high mortality, an adequate antimicrobial therapy starting at an early stage is mandatory. To guide such a therapy, the most promising parameters appear to be plasma levels of procalcitonin (PCT) [4]. Besides PCT, no other sepsis biomarker has achieved universal use throughout different healthcare settings in Germany and neighboring Europe in the last decade. High PCT concentrations are typically found in bacterial infection, in contrast to lower levels in viral infection and levels below 0.1 ng/mL in patients without infection [5]. Furthermore, serum PCT concentrations are positively correlated with the severity of infection. Thus, adequate antibiotic treatment leads to decreasing PCT levels [5].

Recent reviews focused on PCT-based algorithms in patients with infections in general [6,7], respiratory tract infections [8,9], or patients treated in the ICU [10-13]. These reviews focused on different patients in different settings with different treatment algorithms. Most of them included critically ill patients with different disease severity ranging from suspected infection to pneumonia or sepsis [10-13]. The others comprised even more patients of different settings: patients with various diseases such as sepsis, bronchitis, and respiratory tract infection, in various settings (primary care, emergency department, ICU, and inpatient wards) were combined in one review [6,7]. Two reviews focused only on patients with respiratory tract infection [8,9]. However there is no review exclusively including the population of ICU patients with severe sepsis or septic shock: both entities are hereinafter collectively referred to severe sepsis. Our meta-analysis addresses two essential research questions: 1) does a PCT-guided strategy to determine the duration of antimicrobial therapy reduce antibiotic use compared with a strategy not based on PCT? 2) Does such a PCT-guided strategy improve health outcomes compared with a strategy not based on PCT? [14]. As evidence for the effect of PCT-guided therapy in severe sepsis is missing, we investigated the effect of PCT-guided therapy compared to standard care.

## Materials and methods

The systematic review was performed following current guidelines and is reported according to the preferred reporting items for systematic reviews and meta-analyses (PRISMA) statement [15,16].

### Literature search and data extraction

Two reviewers (AP, CW) independently performed a systematic search in the databases Medline via PubMed, Excerpta Medica database (Embase), ISI Web of Knowledge including the databases Web of Science, Journal Citation Reports and Science Citation Index (SCI). Furthermore, searches in BioMed Central, ScienceDirect and the Cochrane Central Register of Controlled Trials were continued. To identify unpublished or ongoing studies and to obtain the study protocols we searched two further websites (http://www.ClinicalTrials.gov and http://www.ISRCTN.org). The following keywords or medical subject headings (MeSH) were used: “procalcitonin” or “PCT” combined with “sepsis” or “SIRS” or “systemic inflammatory response syndrome” or “bacterial infection”. All databases had been searched up to 14 June 2013.

All primary intervention studies were included that compared PCT-guided therapy with standard care according to current guidelines that met the following inclusion criteria: 1) studies that assessed the efficacy of a treatment algorithm based on procalcitonin; 2) studies that had a well-defined standard for the target condition (severe sepsis), which included the use of definitions according to the American College of Chest Physicians (ACCP)/Society of Critical Care Medicine (SCCM) Consensus Conference [17] and the German Sepsis Society [18]; and 3) studies that provided sufficient information to calculate the relative risk (RR) together with 95% CI or the hazard ratio (HR) together with 95% CI respectively.

Eligible studies had to be randomized controlled clinical trials (RCTs) or prospective cohort studies. Studies dealing with neonates were excluded, because of considerable differences in diagnosis, course and therapy of sepsis compared to adults. The published language was not restricted. Disagreements of judgment and inclusion of studies were solved by consensus.

Data were extracted using a structured data collection sheet including the following items: authors and year of study, design, setting, diagnosis, procalcitonin test, randomization, number and characteristics of participants, interventions, outcomes, duration, availability of study protocol, and country. Primary outcomes of this meta-analysis were 28-day mortality and hospital mortality. Secondary outcomes were duration of antimicrobial therapy, length of stay in the ICU and length of stay in the hospital. If additional information was needed, the authors of the studies were contacted by Email. Eligible studies with insufficient data for calculation or missing replies from the authors were excluded.

### Risk of bias

For assessing risk of bias, we applied the Cochrane Collaboration tool for assessing risk of bias by judging seven items representing sources of risk of bias [19]. The following items were evaluated: sequence generation, allocation concealment, blinding of participants and personnel, blinding of outcome assessment, incomplete outcome data, selective reporting, and other bias. The classification of the assessment was: low risk of bias, high risk of bias, or uncertain risk of bias. The assessment was done independently by two reviewers (AP, CW). Disagreements were resolved by consensus.

### Statistical analysis

The statistical calculations were done with the freely available software R [20] using the R package meta [21]. Results were presented graphically using forest plots. As a meta-analysis is an observational study, the statistical analysis covered the investigation of bias and heterogeneity. A formal analysis of publication bias was based on Egger’s test [22]. For dichotomous outcomes (for example, hospital mortality) RRs were calculated for every single study by means of 2 × 2 contingency tables. The calculation of combined estimates was performed using the Mantel-Haenszel method. For continuous outcomes (for example, length of antibiotic treatment) HRs based on the exponential distribution were calculated together with their variances [23]. This approach was chosen because the necessary information for the calculation of standardized mean differences was not available. Thus, the length of stay and antibiotic therapy were modeled using the exponential distribution. This approach was chosen rather than the standardized differences because it can be used if only the mean duration is reported in the respective study. Summary estimates were obtained using the inverse variance method.

Heterogeneity was assessed using the Cochran *Q*-test and the *I*^2^ measure [24]. The heterogeneity variance tau^2^ was calculated using the moment estimator of DerSimonian-Laird [25]. For all outcomes the fixed effect model as well as the random effects model was applied. Outliers and influential studies were investigated using case-deletion techniques analysis in order to identify studies with great impact on the overall results.

## Results

### Literature search

**Figure 1:**
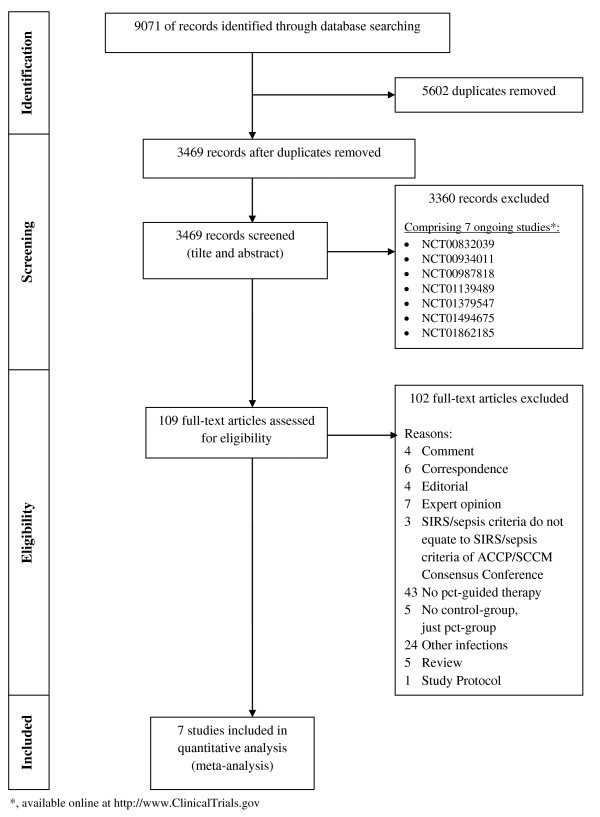
**Preferred reporting items for systematic reviews and meta-analyses** (**PRISMA) flow diagram.** Study identification and selection process. SIRS, systemic inflammatory response syndrome; ACCP, American College of Chest Physicians; SCCM, Society of Critical Care Medicine; PCT, procalcitonin. *, available online at http://www.ClinicalTrials.gov.

We identified 9,071 records by searching the databases PubMed, Embase, ISI Web of Knowledge, BioMed Central, ScienceDirect and the Cochrane Central Register of Controlled Trials. After removing 5,602 duplicates, the titles and abstracts of the remaining 3,469 records were screened. Therefore 3,360 records could be excluded. By assessing the full-text articles of the remaining 109 records, 7 studies [26-32] fulfilled the inclusion criteria and were thus eligible for our meta-analysis (Figure 1). Furthermore, we identified 7 ongoing studies registered at http://www.ClinicalTrials.gov that might deliver important results for future meta-analyses.

We excluded two studies due to insufficient information and missing replies [33,34].

The study from Bagnenko *et al*. [34] was excluded because of missing data and deficits in quality: the inclusion of patients was not clearly described, the exact number of patients treated in each group was not given, for hospital mortality only percentages without absolute numbers were reported, and finally the treatment regimen for the control group was not specified. The authors did not respond to clarify these questions. Another study had to be excluded, because information of a subgroup of 25 patients with sepsis was missing [33]. Again, the authors did not respond to clarify these questions. In particular we tried to obtain the data for all septic patients included in two trials [27,29]. In one case, further information was provided [29], in another the authors did not pass on any further information, so we could only include the patients with septic shock from this study [27].

### Publication bias

As only seven studies were included in the meta-analysis, assessment of publication bias using a funnel plot, followed by a linear regression test of funnel plot asymmetry (Egger’s test) [22], was not possible.

### Study characteristics

All eligible studies were published in the English language. Because no cohort study met the inclusion criteria, the meta-analysis only consists of RCTs. All studies were conducted in Europe between 2003 [32] and 2009 [26]. Four studies were conducted in mixed surgical/medical ICUs [26,27,29,30], and three studies were conducted in surgical ICUs without medical patients [28,31,32]. The number of included patients from each study ranged from 27 [31] to 459 [29] with a total number of 1,075 patients. In two studies the subgroup of septic patients was included [27,29].

For PCT measurements three different PCT tests were used: Brahms PCT Kryptor [26,27,29,30], Brahms PCT LIA [28,31] and Brahms PCT-Q [32]. The main focus in the PCT-guided therapy was on de-escalation in three studies [28,30,31], on de-escalation as well as escalation in two studies [26,27], and mainly on escalation in two studies [29,32]. A summary of study characteristics and treatment algorithms of the eligible studies is shown in Tables 1 and 2.

**Table 1:** Characteristics of included studies

**Study**	**Annane *et al. *** **2013 **[26]	**Bouadma *et al. *** **2010 **[27]	**Hochreiter *et al. *** **2009 **[28]	**Jensen *et al. *** **2011**[29]	**Nobre *et al. *** **2008 **[30]	**Schroeder *et al. *** **2009**[31]	**Svoboda *et al. *** **2007 **[32]
**Design**	RCT	RCT	RCT	RCT	RCT	RCT	RCT
**Setting**	Surgical and medical ICU	Surgical and medical ICU	Surgical ICU	Surgical and medical ICU	Surgical and medical ICU	Surgical ICU	Surgical ICU
**Condition**	Severe sepsis and septic shock	Septic shock	Severe sepsis	Severe sepsis and septic shock	Severe sepsis and septic shock	Severe sepsis	Severe sepsis
**Total number of included patients**	61	267	110	459	79	27	72
**Number of patients**							
PCT group/control group	31/30	138/129 (septic shock)	57/53	247/212	39/40	14/13	38/34
		55/53 (positive blood culture)					
**Hospital mortality**							
Relative risk (95% CI)	0.68 (0.30; 1.55)	NA	1.00 (0.53; 1.86)	NA	1.03 (0.46; 2.31)	0.93 (0.23; 3.81)	NA
Events PCT group/events control group	7/10		15/14		9/9	3/3	
**28-day mortality**							
Relative risk (95% CI)	NA	1.15 (0.81; 1.63)	NA	1.02 (0.80; 1.30)	1.03 (0.43; 2.46)	NA	0.69 (0.35; 1.36)
Events PCT group/events control group		48/39 (septic shock)		90/76	8/8		10/13
**Duration of antibiotic treatment, days**							
PCT group/control group	5/5 (median)	9.8/12.8 (mean) (only positive blood culture)	5.9/7.9 (mean)	NA	6.0/9.5 (median)	6.6/8.3 (mean)	NA
**Length of ICU stay, days**							
PCT group/control group	22/23 (median)	NA	15.5/17.7 (mean)	6.0/5.0 (median)	4.0/7.0 (median)	16.4/16.7 (mean)	16.1/19.4 (mean)
**Length of hospital stay, days**							
PCT group/control group	27/33 (median)	NA	NA	23.0/22.0 (median)	17.0/23.5 (median)	NA	NA
**SOFA score**							
PCT group/control group	9.5/10 (median)	NA	6.7/7.0 (mean)	NA	6.4/6.6 (mean)	7.3/8.3 (mean)	7.9/9.3 (mean)
	8.5 to 11/8 to 11 (IQR)		3.68/3.62 (SD)		3.3/3.0 (SD)	3.5/4.2 (SD)	2.8/3.3 (SD)
Medical patients, %*	97%	89%	0%	59%	NA	0%	0%
**Subgroup of study**	No	Yes	No	Yes	No	No	No
**Duration of study, months**	36	12	15	29	15	7	29
**Study protocol available**	Yes	Yes	Yes	Yes	Yes	No	No
**Country**	France	France	Germany	Denmark	Switzerland	Germany	Czech Republic

PCT, procalcitonin; RCT, randomized controlled clinical trial; SOFA, sequential organ failure assessment; NA, data were not available; *data were stated in study or calculated from information given in study.

**Table 2:** PCT assays and algorithms used for procalcitonin (PCT)-guided treatment in the included studies

**Study**	**PCT test**	**Regimen in the PCT group**	**Regimen in the control group**
**Annane *et al. *** **2013**[26]	Brahms PCT Kryptor	**Medical patients:**	Antibiotic treatment at the discretion of the patient’s physician
		PCT <0.25 ng/mL: antibiotics not initiated or stopped	
		PCT ≥ 0.25 and < 0.5 ng/mL: Antibiotics strongly discouraged	
		PCT ≥0.5 and <5 ng/mL: antibiotics recommended	
		PCT ≥5 ng/mL: antibiotics strongly recommended	
		**Surgical patients:**	
		PCT <4 ng/mL: antibiotics not initiated or stopped	
		PCT ≥4 and <9 ng/mL: antibiotics recommended	
		PCT ≥9 ng/mL: antibiotics strongly recommended	
**Bouadma *et al. *** **2010 **[27]	Brahms PCT Kryptor	**Guidelines for starting of antibiotics:**	Treatment according to international and local guidelines
		PCT <0.25 ng/mL: antibiotics strongly discouraged	
		PCT ≥0.25 and <0.5 ng/mL: antibiotics discouraged	
		PCT ≥0.5 and <1 ng/mL: antibiotics encouraged	
		PCT ≥1 ng/mL: antibiotics strongly encouraged	
		**Guidelines for continuing or stopping of antibiotics:**	
		PCT <0.25 ng/mL: stopping of antibiotics strongly encouraged.	
		Decrease by ≥80% from peak concentration, or concentration ≥0.25 and <0.5 ng/mL: stopping of antibiotics encouraged	
		Decrease by <80% from peak concentration and concentration ≥0.5 ng/mL: continuing of antibiotics encouraged	
		Increase of concentration compared with peak concentration and concentration ≥0.5 ng/mL: changing of antibiotics strongly encouraged	
**Hochreiter *et al. *** **2009 **[28]	Brahms PCT LIA	PCT < 1 ng/mL: Antibiotics discontinued.	Antibiotic treatment according to standard regimen over 8 days
		PCT >1 ng/mL and dropped to 25 to 35% of the initial value over 3 days: antibiotics discontinued	
		Additionally the infection had to improve clinically	
**Jensen *et al. *** **2011 **[29]	Brahms PCT Kryptor	Single baseline measurement of PCT ≥1.00 ng/mL or PCT ≥1.00 ng/mL and not decreased at least 10% from the previous day:	Antibiotic treatment according to current guidelines
		1) increasing the antimicrobial spectrum covered	
		2) intensifying the diagnostic effort to find uncontrolled sources of infection	
		PCT <1.00 ng/mL for at least 3 days: de-escalation possible	
**Nobre *et al. *** **2008**[30]	Brahms PCT Kryptor	Patients with PCT <1 ng/mL re-evaluated at day 3: antibiotics discontinued if PCT <0.1 ng/mL	Antibiotic treatment based on empirical rules
		Patients with PCT ≥1 ng/mL re-evaluated at day 5: antibiotics discontinued if PCT dropped >90% from the baseline peak level or if PCT <0,25 ng/mL	
**Schroeder *et al. *** **2009 **[31]	Brahms PCT LIA	PCT <1 ng/mL and clinical signs of infection improved: antibiotics discontinued	Antibiotic treatment according to clinical signs and empiric rules
		PCT dropped to <35% of the initial concentration within 3 days and clinical signs of infection improved: antibiotics discontinued	
**Svoboda *et al. *** **2007 **[32]	Brahms PCT-Q	PCT >2 ng/mL: change of antibiotics and catheters	Treatment according to contemporary treatment protocol of the institute
		PCT ≤2 ng/mL: ultrasonography and/or computer tomography followed by repeated surgical treatment if localized infection was confirmed	

PCT, procalcitonin.

### Risk of bias

The overall risk of bias was moderate according to the Cochrane Collaboration tool for assessing risk of bias of all included studies. Two studies achieved low overall risk of bias [27,32], whereas the remaining studies had moderate overall risk of bias [26,28-31]. Two items had low risk of bias in all studies: “incomplete outcome data” and “other bias”. Oftentimes the risk of bias remained unclear due to insufficient information given in each study.

**Figure 2:**
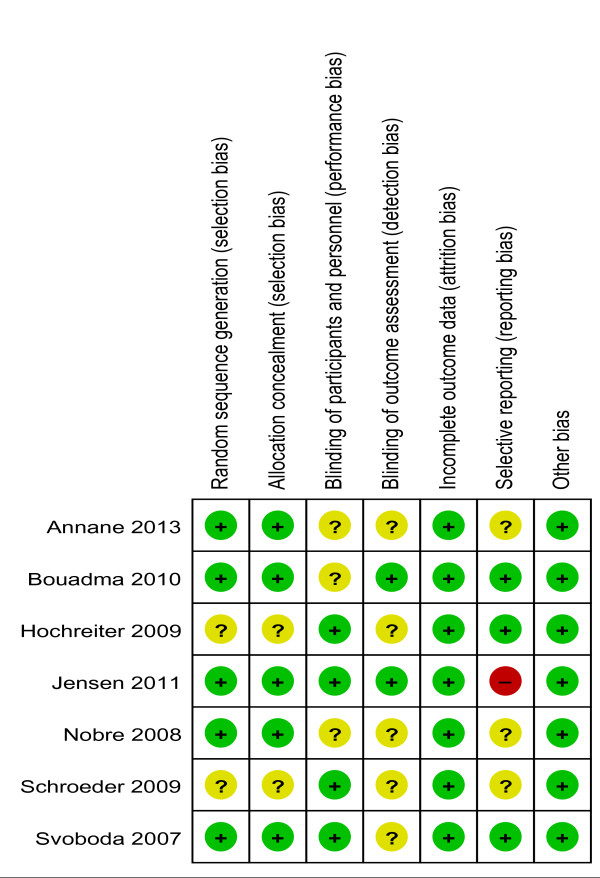
**Risk of bias summary.** Cochrane Collaboration tool for assessing risk of bias. Review authors’ judgments about each risk of bias item for each included study. +, low risk of bias; -, high risk of bias; ?, unclear risk of bias.

Figure 2 summarizes the risk of bias of included studies. Detailed information about risk of bias, support for judgment of bias and graphically summarized information on risk of bias are given in the online supplement (Additional files 1 and 2).

### Combined estimates

#### Primary outcomes

**Figure 3:**
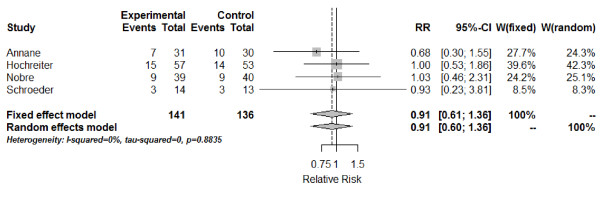
**Forest plot - hospital mortality.** The forest plot represents the relative risk (RR) together with the 95% CI comparing patients treated in the procalcitonin (PCT) and the control groups (Control). Events, number of deceased patients in group; Experimental, PCT group; Total, number of all patients in group; W, weight of individual studies (in fixed- and random-effects model).

Hospital mortality in severe sepsis patients was reported in four studies [26,28,30,31]. The combined estimate of the RR based on the fixed-effect model for hospital mortality is 0.91 (95% CI: 0.61; 1.36) (Figure 3), with no differences between the PCT-guided therapy and standard-care group. The test of heterogeneity showed no significant heterogeneity between these studies (*Q* = 0.66; df = 3; *I*^2^ = 0%; tau^2^ = 0; *P* = 0.8835).

**Figure 4:**
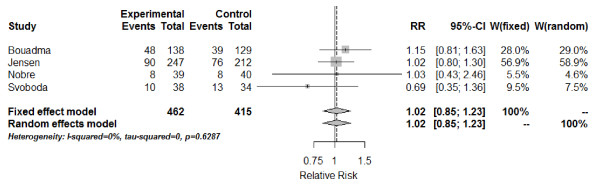
**Forest plot - 28-day mortality.** The forest plot represents the relative risk (RR) together with the 95% CI comparing patients treated in the procalcitonin (PCT) and the control groups (Control). Events, number of deceased patients in group; Experimental, PCT group; Total, number of all patients in group; W, weight of individual studies (in fixed- and random-effects model).

The 28-day mortality was covered in four studies [27,29,30,32]. The combined estimate of the RR based on the fixed-effect model for 28-day mortality is 1.02 (95% CI: 0.85; 1.23) (Figure 4), again, with no difference between the PCT-guided therapy and standard care group. The test of heterogeneity showed no significant heterogeneity between these studies (*Q* = 1.74; df = 3; *I*^2^ = 0%; tau^2^ = 0; *P* = 0.6287).

The influential analysis using both the fixed-effect model and the random-effects model showed that no study had great impact on the overall results of hospital mortality and 28-day mortality, respectively.

#### Secondary outcomes

**Figure 5:**
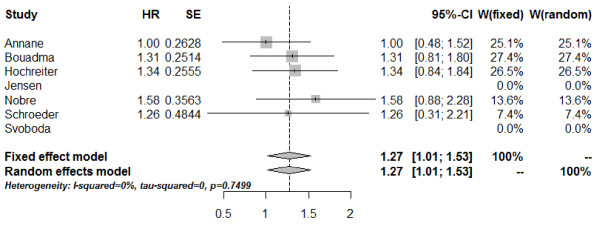
**Forest plot - duration of antimicrobial therapy.** The forest plot represents the hazard ratios (HRs) together with the 95% CIs comparing patients treated in the procalcitonin (PCT) and the control groups. SE, standard error; W, weight of individual studies (in fixed- and random-effects model).

The duration of antimicrobial therapy in severe sepsis patients was documented in five studies [26-28,30,31]. In one study we could only include the subgroup of septic patients with positive blood cultures for assessment of this outcome [27]. The combined estimate for the duration of antimicrobial therapy assessed as the HR and based on the fixed-effect model amounted to 1.27 (95% CI: 1.01; 1.53) (Figure 5). These results indicate a significantly shorter median duration of antimicrobial therapy with PCT-guided therapy compared to standard therapy. No significant heterogeneity could be detected between these studies (*Q* = 1.92; df = 4; I^2^ = 0%; tau^2^ = 0; *P* = 0.7499).

**Figure 6:**
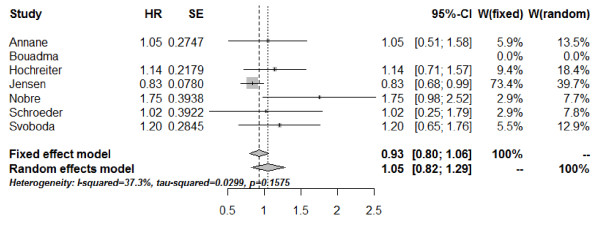
**Forest plot - length of stay in the ICU.** The forest plot represents the hazard ratios (HRs) together with the 95% CIs comparing patients treated in the procalcitonin (PCT) and the control groups. SE, standard error; W, weight of individual studies (in fixed- and random-effects model).

The length of stay in the ICU (ICU-LOS) was documented in five studies [26,28,30-32]. Additionally, information on ICU-LOS for the subgroup of patients with severe sepsis of the study from Jensen *et al*. [29] was available by correspondence (median ICU-LOS of 6 days in the PCT group versus 5 days in the control group). The combined estimate for ICU-LOS assessed as the HR and based on the fixed-effect model is 0.93 (95% CI: 0.80; 1.06) (Figure 6). No significant heterogeneity was detected between these studies (*Q* = 7.98; df = 5; *I*^2^ = 37.3%; tau^2^ = 0.0227; *P* = 0.1575).

**Figure 7:**
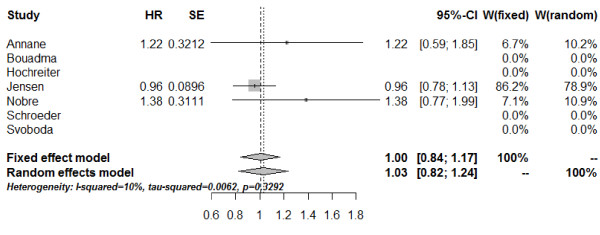
**Forest plot - length of stay in the hospital.** The forest plot represents the hazard ratios (HRs) together with the 95% CIs comparing patients treated in the procalcitonin (PCT) and the control groups. SE, standard error; W, weight of individual studies (in fixed- and random-effects model).

The length of hospital stay was reported in two studies [26,30]. In addition we obtained the median hospital stay for the subgroup of patients with severe sepsis of the study from Jensen *et al*. [29] by correspondence with the author (23 days in the PCT group versus 22 days in the control group). The combined estimate assessed by the HR and fixed-effect model amounted 1.00 (95% CI: 0.84; 1.17) (Figure 7). No significant heterogeneity was detected between these studies (*Q* = 2.22; df = 2; *I*^2^ = 10%; tau^2^ = 0.0069; *P* = 0.3292).

The influential analysis using the fixed-effect model showed no predominant influence on the combined HR of duration of antimicrobial therapy in any study. As shown in the influential analyses based on the fixed-effect model, the study from Jensen *et al*. had great influence on the combined estimates of ICU-LOS and length of hospital stay, respectively. When omitting this study, the results show a trend towards shorter ICU-LOS (HR: 1.19; 95% CI: 0.93; 1.44) and hospital stay (HR: 1.30; 95% CI: 0.87; 1.74) with PCT-guided therapy.

We did not detect significant heterogeneity among these studies and therefore did not perform a meta-regression analysis. Furthermore, the data basis for a meta-regression is rather small.

## Discussion

Our meta-analysis aimed to investigate the impact of a PCT-guided therapy compared to standard treatment administered to severe sepsis patients treated in an ICU. Contrary to previous reviews that analyzed patients in various settings with different disease severities, we focused on patients with severe sepsis, a population in which clinical decision-making to stop antibiotic treatment is challenging. In view of the high mortality rate in severe sepsis, clinicians believe themselves to “be on the safer side” with more prolonged courses of antimicrobial treatment. Current evidence to limit duration of antibiotic treatment to 7 to 10 days is rather low and has been included only as a grade-2C recommendation in the recent guidelines of the Surviving Sepsis Campaign [35], in which the authors state that “decisions to continue, narrow, or stop antimicrobial therapy must be made on the basis of clinician judgment and clinical information”. Variance of treatment regimens in the control groups of our meta-analysis might therefore reflect the current way of treatment of patients with severe sepsis.

Our findings do not show a significant difference between a PCT-guided therapy and standard care treatment regarding 28-day or hospital mortality, respectively. Furthermore, length of stay in the ICU and in-hospital stay were not different between both groups. However, we found a significant reduction of the length of antibiotic therapy in favor of a PCT-guided therapy strategy. Our calculations, based on the exponential model, indicate a median length of antibiotic treatment of 6 days in the PCT-guided group compared to 8 days in the control group, resulting in a median reduction of approximately 2 days. A reduction of antimicrobial therapy using biomarkers for clinical decision-making may have certain advantages, as antimicrobial resistance becomes more prevalent by using prolonged courses of broad-spectrum antimicrobial agents for treatment of severe sepsis patients [36,37]. Moreover, antibiotic consumption and acquired antimicrobial resistance had been shown to be associated with increased mortality, morbidity, length of hospitalization, and health care costs [38,39].

Currently, no treatment algorithms for guidance of severe sepsis treatment using PCT levels are well established in this high-risk population. To establish a PCT-guided treatment algorithm in severe sepsis patients, it is important to distinguish between escalation and de-escalation of therapeutic interventions. Reliable cut-off values of PCT levels to guide therapeutic decisions need to be defined in future studies, since treatment algorithms varied substantially between the studies included in our systematic review. For instance, Annane *et al*. and Bouadma *et al*. [26,27] encouraged a prolongation of antibiotic treatment if PCT levels were above 0.5 ng/mL, and discouraged antibiotic treatment if levels dropped below 0.5 ng/mL. Furthermore, Annane *et al*. distinguished between medical and surgical patients. In surgical patients a different algorithm was applied with a recommendation to stop antibiotics when PCT levels were below 4 ng/mL. Bouadma *et al*. recommended stopping antibiotics at PCT levels below 0.25 ng/mL. If PCT levels were between 0.5 ng/mL and 0.25 ng/mL, antibiotics were stopped if there was a decrease of at least 80% of the peak concentration of PCT levels. Four studies recommended a PCT level of 1.0 ng/mL as the cutoff [28-31]. Hochreiter *et al*. and Schroeder *et al*. recommended discontinuing antibiotics if PCT was below 1.0 ng/mL. Both recommended to discontinue antibiotics if PCT levels dropped by 35% of the initial level within 3 days. Nobre *et al*. recommended discontinuing antibiotics in patients with PCT levels below 0.1 ng/mL after 3 days. In patients with PCT levels above 1.0 ng/mL antibiotics were discontinued if PCT levels dropped more than 90% from the baseline peak level or if PCT levels were below 0.25 ng/mL after 5 days.

In contrast to all other studies, Jensen *et al*. tested a rigorous escalation strategy [29]. In the case of PCT levels above 1.0 ng/mL an intensified antibiotic treatment strategy was recommended. De-escalation was only possible if PCT levels dropped below 1.0 ng/mL for at least 3 days. This treatment algorithm led to a prolonged length of stay in the ICU and substantially higher use of broad-spectrum antimicrobials. A different cut-off level of 2.0 ng/mL was used by Svoboda *et al*. in surgical patients [32].

This meta-analysis has several limitations. First, studies included in our meta-analysis varied substantially in study design and objectives. Two studies provided rules for starting or continuing as well as discontinuing antibiotic treatment [26,27]. Three studies focused on de-escalation of antibiotic therapy [28,30,31], whereas two studies placed the focus on escalation of antibiotic treatment and diagnostic efforts [29,32] and surgical treatment [32]. Second, a limited number of patients is included, third, a combined analysis of medical and surgical patients is problematic, since there are substantial differences in outcomes regarding surgical patients, where surgical source-control measures play a dominant role.

In order to explore knowledge on a PCT-guided treatment in severe sepsis patients in more detail, we await the results of seven ongoing studies registered at http://www.ClinicalTrials.gov that might deliver important results for future systematic reviews.

## Conclusion

An approach along a biomarker-guided treatment algorithm using procalcitonin levels may be helpful to guide antimicrobial treatment in severe sepsis patients, treated in ICUs and reduces the duration of antimicrobial therapy without an obvious increase in mortality. However, more research is urgently needed to investigate the safety and effectiveness in subgroups of surgical and medical severe sepsis patients, treated in ICUs. Most importantly, treatment algorithms differ substantially and have to be clarified in future studies.

## Key messages

• A PCT-guided treatment reduces the duration of antimicrobial therapy in severe sepsis patients, without increasing 28-day and in-hospital mortality rates

• Recommendations for PCT-guided treatment algorithms for treatment of severe sepsis patients differ substantially among published studies

• Future studies have to show which PCT-guided treatment algorithms could be recommended in severe sepsis patients

## Abbreviations

Df: Degrees of freedom; HR: Hazard ratio; I2: Percentage of variation across studies that is due to heterogeneity rather than chance; ICU-LOS: Length of stay in the ICU; PCT: Procalcitonin; PRISMA: Preferred reporting items for systematic reviews and meta-analyses; Q: Heterogeneity statistic; RCT: Randomized controlled clinical trial; RR: Relative risk; SE: Standard error; SIRS: Systemic inflammatory response syndrome; tau: Square-root of between-study variance (moment estimator of DerSimonian-Laird); W: Weight of individual studies (in fixed- and random-effects model); WMD: Weighted mean difference.

## Competing interests

The authors declare that they have no competing interests.

## Authors’ contributions

AP conceived and designed the study, did the literature search and the acquisition of data, analyzed and interpreted data, drafted and critically revised the manuscript for important intellectual content. CW did the literature search and the acquisition of data, drafted and critically revised the manuscript for important intellectual content. FMB conceived and designed the study, drafted and critically revised the manuscript for important intellectual content, supervised the study and gave administrative, technical or material support. PS conceived and designed the study, statistically analyzed and interpreted the data, drafted and critically revised the manuscript for important intellectual content, supervised the study and gave administrative, technical or material support. All authors read and approved the final version of the manuscript.

## Supplementary Material

Additional file 1 **Risk of bias table.** Cochrane Collaboration tool for assessing risk of bias. Detailed information about risk of bias and support for judgment of bias. Click here for file

Additional file 2 **Risk of bias graph.** Cochrane Collaboration tool for assessing risk of bias. Review authors’ judgments about each risk of bias item presented as percentages across all included studies. Click here for file

## References

[B1] Engel C Brunkhorst FM Bone HG Brunkhorst R Gerlach H Grond S Gruendling M Huhle G Jaschinski U John S Mayer K Oppert M Olthoff D Quintel M Ragaller M Rossaint R Stuber F Weiler N Welte T Bogatsch H Hartog C Loeffler M Reinhart K Epidemiology of sepsis in Germany: results from a national prospective multicenter study Intensive Care Med 2007 17 606 618 10.1007/s00134-006-0517-7 17323051

[B2] Brunbuisson C Doyon F Carlet J Dellamonica P Gouin F Lepoutre A Mercier JC Offenstadt G Regnier B Incidence, risk-factors, and outcome of severe sepsis and septic shock in adults - a multicenter prospective-study in intensive-care units JAMA 1995 17 968 974 10.1001/jama.1995.03530120060042 7674528

[B3] Heublein S Hartmann M Hagel S Hutagalung R Brunkhorst FM Epidemiology of sepsis in German hospitals derived from administrative databases Infection 2013 17 S71

[B4] Carrol ED Thomson AP Hart CA Procalcitonin as a marker of sepsis Int J Antimicrob Agents 2002 17 1 9 12127705 10.1016/s0924-8579(02)00047-x

[B5] Assicot M Gendrel D Carsin H Raymond J Guilbaud J Bohuon C High serum procalcitonin concentrations in patients with sepsis and infection Lancet 1993 17 515 518 10.1016/0140-6736(93)90277-N 8094770 PMC7141580

[B6] Schuetz P Chiappa V Briel M Greenwald JL Procalcitonin algorithms for antibiotic therapy decisions: a systematic review of randomized controlled trials and recommendations for clinical algorithms Arch Intern Med 2011 17 1322 1331 10.1001/archinternmed.2011.318 21824946

[B7] Tang H Huang T Jing J Shen H Cui W Effect of Procalcitonin-Guided Treatment in Patients with Infections: a Systematic Review and Meta-Analysis Infection 2009 17 497 507 10.1007/s15010-009-9034-2 19826761

[B8] Schuetz P Briel M Christ-Crain M Stolz D Bouadma L Wolff M Luyt CE Chastre J Tubach F Kristoffersen KB Wei L Burkhardt O Welte T Schroeder S Nobre V Tamm M Bhatnagar N Bucher HC Mueller B Procalcitonin to guide initiation and duration of antibiotic treatment in acute respiratory infections: an individual patient data meta-analysis Clin Infect Dis 2012 17 651 662 10.1093/cid/cis464 22573847 PMC3412690

[B9] Zhang L Huang J Xu T Lin Y Procalcitonin-guided algorithms of antibiotic therapy in community-acquired lower respiratory tract infections: a systematic review and meta-analysis of randomized controlled trials [article in Chinese] Zhonghua Jie He He Hu Xi Za Zhi 2012 17 275 282 22781200

[B10] Heyland DK Johnson AP Reynolds SC Muscedere J Procalcitonin for reduced antibiotic exposure in the critical care setting: A systematic review and an economic evaluation Crit Care Med 2011 17 1792 1799 10.1097/CCM.0b013e31821201a5 21358400

[B11] Kopterides P Siempos II Tsangaris I Tsantes A Armaganidis A Procalcitonin-guided algorithms of antibiotic therapy in the intensive care unit: A systematic review and meta-analysis of randomized controlled trials Crit Care Med 2010 17 2229 2241 10.1097/CCM.0b013e3181f17bf9 20729729

[B12] Matthaiou DK Ntani G Kontogiorgi M Poulakou G Armaganidis A Dimopoulos G An ESICM systematic review and meta-analysis of procalcitonin-guided antibiotic therapy algorithms in adult critically ill patients Intensive Care Med 2012 17 940 949 10.1007/s00134-012-2563-7 22538461

[B13] Agarwal R Schwartz DN Procalcitonin to guide duration of antimicrobial therapy in intensive care units: a systematic review Clin Infect Dis 2011 17 379 387 10.1093/cid/cir408 21810753

[B14] Noorani HZ Adams E Pitrak D Belinson S Aronson N Future Research Needs on Procalcitonin-Guided Antibiotic Therapy. Future Research Needs Paper No. 29 http://www.effectivehealthcare.ahrq.gov/ehc/products/497/1377/FRN29_Procalcitonin_FinalReport_20130109.pdf 24783273

[B15] Liberati A Altman DG Tetzlaff J Mulrow C Gotzsche PC Ioannidis JP Clarke M Devereaux PJ Kleijnen J Moher D The PRISMA statement for reporting systematic reviews and meta-analyses of studies that evaluate health care interventions: explanation and elaboration Ann Intern Med 2009 17 W65 W94 19622512 10.7326/0003-4819-151-4-200908180-00136

[B16] Moher D Liberati A Tetzlaff J Altman DG Preferred reporting items for systematic reviews and meta-analyses: the PRISMA statement Ann Intern Med 2009 17 264 269 10.7326/0003-4819-151-4-200908180-00135 19622511

[B17] American College of Chest Physicians/Society of Critical Care Medicine Consensus Conference Definitions for sepsis and organ failure and guidelines for the use of innovative therapies in sepsis Crit Care Med 1992 17 864 874 10.1097/00003246-199206000-00025 1597042

[B18] Reinhart K Brunkhorst FM Bone HG Bardutzky J Dempfle CE Forst H Gastmeier P Gerlach H Grundling M John S Kern W Kreymann G Kruger W Kujath P Marggraf G Martin J Mayer K Meier-Hellmann A Oppert M Putensen C Quintel M Ragaller M Rossaint R Seifert H Spies C Stuber F Weiler N Weimann A Werdan K Welte T Prevention, diagnosis, treatment, and follow-up care of sepsis - First revision of the S2k Guidelines of the German Sepsis Society (DSG) and the German Interdisciplinary Association for Intensive and Emergency Care Medicine (DIVI) Anaesthesist 2010 17 347 370 10.1007/s00101-010-1719-5 20414762

[B19] Higgins JPT Altman DG Sterne JAC Higgins JPT, Green S Chapter 8: Assessing risk of bias in included studies Cochrane Handbook for Systematic Reviews of Interventions Version 5.1.0 (updated March 2011) http://www.cochrane-handbook.org

[B20] R Development Core Team R: A Language and Environment for Statistical Computing http://www.R-project.org/

[B21] Schwarzer G Meta: Meta-Analysis with R, R Package Version: 1.6-1 http://CRAN.R-project.org/package=meta

[B22] Deeks JJ Macaskill P Irwig L The performance of tests of publication bias and other sample size effects in systematic reviews of diagnostic test accuracy was assessed J Clin Epidemiol 2005 17 882 893 10.1016/j.jclinepi.2005.01.016 16085191

[B23] Collet D Modelling Survival Data in Medical Research 2003 2 Boca Raton (FL): CRC Press

[B24] Higgins JP Thompson SG Deeks JJ Altman DG Measuring inconsistency in meta-analyses BMJ 2003 17 557 560 10.1136/bmj.327.7414.557 12958120 PMC192859

[B25] DerSimonian R Laird N Meta-analysis in clinical trials Control Clin Trials 1986 17 177 188 10.1016/0197-2456(86)90046-2 3802833

[B26] Annane D Maxime V Faller JP Mezher C Clec’h C Martel P Gonzales H Feissel M Cohen Y Capellier G Gharbi M Nardi O Procalcitonin levels to guide antibiotic therapy in adults with non-microbiologically proven apparent severe sepsis: a randomised controlled trial BMJ Open 2013 17 e002186 doi:10.1136/bmjopen-2012-00218doi:10.1136/bmjopen-2012-00218 23418298 10.1136/bmjopen-2012-002186 PMC3586059

[B27] Bouadma L Luyt CE Tubach F Cracco C Alvarez A Schwebel C Schortgen F Lasocki S Veber B Dehoux M Bernard M Pasquet B Regnier B Brun-Buisson C Chastre J Wolff M Use of procalcitonin to reduce patients’ exposure to antibiotics in intensive care units (PRORATA trial): a multicentre randomised controlled trial Lancet 2010 17 463 474 10.1016/S0140-6736(09)61879-1 20097417

[B28] Hochreiter M Koehler T Schweiger AM Keck FS Bein B von Spiegel T Schroeder S Procalcitonin to guide duration of antibiotic therapy in intensive care patients: a randomized prospective controlled trial Crit Care 2009 17 R83 10.1186/cc7903 19493352 PMC2717450

[B29] Jensen JU Hein L Lundgren B Bestle MH Mohr TT Andersen MH Thornberg KJ Loken J Steensen M Fox Z Tousi H Soe-Jensen P Lauritsen AO Strange D Petersen PL Reiter N Hestad S Thormar K Fjeldborg P Larsen KM Drenck NE Ostergaard C Kjaer J Grarup J Lundgren JD Procalcitonin-guided interventions against infections to increase early appropriate antibiotics and improve survival in the intensive care unit: A randomized trial Crit Care Med 2011 17 2048 2058 10.1097/CCM.0b013e31821e8791 21572328

[B30] Nobre V Harbarth S Graf L-D Rohner P Pugin J Use of procalcitonin to shorten antibiotic treatment duration in septic patients - A randomized trial Am J Respir Crit Care Med 2008 17 498 505 10.1164/rccm.200708-1238OC 18096708

[B31] Schroeder S Hochreiter M Koehler T Schweiger AM Bein B Keck FS von Spiegel T Procalcitonin (PCT)-guided algorithm reduces length of antibiotic treatment in surgical intensive care patients with severe sepsis: results of a prospective randomized study Langenbecks Arch Surg 2009 17 221 226 10.1007/s00423-008-0432-1 19034493

[B32] Svoboda P Kantorova I Scheer P Radvanova J Radvan M Can procalcitonin help us in timing of re-intervention in septic patients after multiple trauma or major surgery? Hepatogastroenterology 2007 17 359 363 17523274

[B33] Stolz D Smyrnios N Eggimann P Pargger H Thakkar N Siegemund M Marsch S Azzola A Rakic J Mueller B Tamm M Procalcitonin for reduced antibiotic exposure in ventilator-associated pneumonia: a randomised study Eur Respir J 2009 17 1364 1375 10.1183/09031936.00053209 19797133

[B34] Bagnenko SF Shliapnikov SA Korol’kov AI Cholangitis and biliary sepsis: problem and ways of solution Vestn Khir Im I I Grek 2009 17 17 20 19663273

[B35] Dellinger RP Levy MM Rhodes A Annane D Gerlach H Opal SM Sevransky JE Sprung CL Douglas IS Jaeschke R Osborn TM Nunnally ME Townsend SR Reinhart K Kleinpell RM Angus DC Deutschman CS Machado FR Rubenfeld GD Webb SA Beale RJ Vincent JL Moreno R Surviving Sepsis Campaign Guidelines Committee including the Pediatric Subgroup. Surviving sepsis campaign: international guidelines for management of severe sepsis and septic shock: 2012 Crit Care Med 2013 17 580 637 10.1097/CCM.0b013e31827e83af 23353941

[B36] Goldmann DA Weinstein RA Wenzel RP Tablan OC Duma RJ Gaynes RP Schlosser J Martone WJ Strategies to prevent and control the emergence and spread of antimicrobial-resistant microorganisms in hospitals - A challenge to hospital leadership JAMA 1996 17 234 240 10.1001/jama.1996.03530270074035 8604178

[B37] Goossens H Antibiotic consumption and link to resistance Clin Microbiol Infect 2009 17 12 15 19366364 10.1111/j.1469-0691.2009.02725.x

[B38] Cosgrove SE The relationship between antimicrobial resistance and patient outcomes: Mortality, length of hospital stay, and health care costs Clin Infect Dis 2006 17 S82 S89 10.1086/499406 16355321

[B39] Roberts RR Hota B Ahmad I Scott RD II Foster SD Abbasi F Schabowski S Kampe LM Ciavarella GG Supino M Naples J Cordell R Levy SB Weinstein RA Hospital and Societal Costs of Antimicrobial-Resistant Infections in a Chicago Teaching Hospital: Implications for Antibiotic Stewardship Clin Infect Dis 2009 17 1175 1184 10.1086/605630 19739972

