# Supplementary material for: Procalcitonin-guided therapy in intensive care unit patients with severe sepsis and septic shock – a systematic review and meta-analysis
Source: Crit Care. 2013 Dec 11;17(6):R291. doi: 10.1186/cc13157 (PMC4056085; doi:10.1186/cc13157)
Supplement: Additional file 1 — Risk of bias table. Cochrane Collaboration tool for assessing risk of bias. Detailed information about risk of bias and support for judgment of bias. [file cc13157-S1.pdf]

Additional file 1. Risk of Bias Table
Judgment of bias via Cochrane Collaboration's tool for assessing risk of bias

Study: Annane et al. 2013

Bias	Authors´ Judgment	Support for Judgment
Random Sequence Generation (Selection Bias)	Low risk	**Quote:** "Patients were randomised in a 1:1 ratio according to a computer-generated list. Randomisation was centralised through a secured website and performed by an independent statistician, and was stratified by the centre and according to whether or not patients underwent surgery in the past 48 h, using permutation blocks, the size of which remained unknown to the investigators."
Allocation Concealment (Selection Bias)	Low risk	**Quote:** "Randomisation was centralised through a secured website and performed by an independent statistician, and was stratified by the centre and according to whether or not patients underwent surgery in the past 48 h, using permutation blocks, the size of which remained unknown to the investigators."
Blinding of Participants and Personnel (Performance Bias)	Unclear risk	**Quote:** "Masking of antibiotic therapy was not feasible in this study. In the control arm, patients, physicians, nurses, investigators, study coordinators, the statistician and the sponsor remained blinded to PCT levels throughout the study."<br>**Quote:** "In the control arm, the decision to start or stop antibiotic therapy was at the discretion of the patient's physician, without knowledge of the patient's PCT concentrations."<br>**Quote:** "In the experimental arm, both initiation and discontinuation of antibiotics were guided by a PCT-based algorithm, applied at 6 h and on day 3 and day 5 postrandomisation."<br>**Quote:** "Investigators were strongly asked not to over-rule the algorithm every day up to the study day 5."<br>**Quote:** "n the experimental arm, physicians were noncompliant with the PCT-based algorithm in 19% of patients at 6 h, 17% on day 3 and 37% on day 5."<br>**Comment:** Patients treated according to a strict protocol but physicians were non-compliant with the algorithms in a notable amount of cases.
Blinding of Outcome Assessment (Detection Bias)	Unclear risk	**Comment:** No information given.
Incomplete Outcome Data (Attrition Bias)	Low risk	**Quote:** "From December 2006 to December 2009, of 1250 patients with the phenotype of severe sepsis or septic shock screened in the eight participating centres, only 62 patients were eligible."<br>**Quote:** "Of the 62 were randomised patients, 31 to each arm, 4 (3 in the control arm and 1 in the

		experimental arm) later withdrew their consent. According to French regulations, vital status being public information, mortality data are given for all randomised patients.”<br>**Comment:** Just few exclusions in both groups. Same reason for missing outcome data across groups.
Selective Reporting (Reporting Bias)	Unclear risk	**Primary outcomes mentioned in protocol and mentioned in published article:**<br>- rate of patients undergoing antibiotic treatment at day 5 postrandomisation<br>**Secondary outcomes mentioned in protocol and mentioned in published article:**<br>- evolution of the SOFA score between day 0, day 3 and day 5<br>**Secondary outcomes mentioned in published article and not mentioned in protocol:**<br>- death at day 5, at ICU discharge and at hospital discharge<br>- proportion of patients started on antibiotics postrandomisation<br>- duration of antibiotic exposure<br>- proportion of patients with infection acquired between randomisation and day 3, day 5 and ICU discharge<br>- ICU length-of-stay<br>- hospital length-of-stay<br>**Comment:** In the published article there are many secondary outcomes mentioned which are not part of the protocol.<br>**Registration of study:** http://www.Clinicaltrials.gov: NCT01025180
Other Bias	Low risk	**Quote:** “This work was partly funded by Thermo Fisher B.R.A.H.M.S. France, a subsidiary of the maker of the PCT assay used in this study. The sponsor has no input in study design, conduct and reporting. It helped with logistic support when organising investigators meeting.”<br>**Quote:** “Competing interests: None.”<br>**Quote:** “The two groups were well balanced for demographic and anthropometric characteristics, the prevalence and severity of co-morbidities and the severity of acute illness.”<br>**Comment:** No extreme baseline imbalance between groups.

Study: Bouadma et al. 2010

Bias	Authors´ Judgment	Support for Judgment
Random Sequence Generation (Selection Bias)	Low risk	**Quote:** "After baseline screening, an independent, centralised, computer-generated randomisation sequence (CleanWeb, Télémedecine, Technologies, Boulogne, France) was used to randomly assign patients in a 1:1 ratio to the procalcitonin or control groups. Patients were stratified by centre with random block sizes of 2, 4, or 6"
Allocation Concealment (Selection Bias)	Low risk	**Quote:** "Patients were stratified by centre with random block sizes of 2, 4, or 6; investigators were masked to assignment before, but not after, randomisation, as per our open-label design. This system was password protected and accessed by the principal investigator or study coordinator after the patient or surrogate gave consent and had met inclusion criteria. The patient's initials and date of birth were entered and then the patient's allocation was assigned."
Blinding of Participants and Personnel (Performance Bias)	Unclear risk	**Comment:** Patients treated according to a strict protocol, but final decision was up to the patient´s physician, irrespective of the procalcitonin concentration.<br>**Quote:** "Investigators used predefined algorithms to guide physicians to start or discontinue antibiotics according to serum procalcitonin concentrations, using a modified version of a previously published algorithm."<br>**Quote:** "Additionally, the final decision with respect to starting and continuing of antibiotics was at the discretion of the patients' physicians, irrespective of the procalcitonin concentration. "<br>**Quote:** "For patients in the control group, before study onset all investigators received and approved a reminder including recommendations for duration of antimicrobial treatment for the most frequent infections (webappendix pp 1–3); these recommendations were derived from international and local guidelines."<br>**Quote:** "Recommendations about duration of antimicrobial treatment for the procalcitonin group were not followed in 219 episodes."<br>**Quote:** „Recommendations about duration of antimicrobial treatment for the control group were not heeded in 146 episodes"<br>**Quote:** „53% of patients randomised to the procalcitonin group were not given algorithm-guided treatment, either because the algorithm was overruled (physicians refused to start or stop antibiotics, even though the algorithm recommended it), or because they were discharged from the intensive care unit, precluding serial serum procalcitonin measurements."

Blinding of Outcome Assessment (Detection Bias)	Low risk	**Quote:** "Although treatment assignments were not masked, all investigators were unaware of aggregate outcomes during the study, and primary endpoints were strictly defined and not patient-reported."
Incomplete Outcome Data (Attrition Bias)	Low risk	**Quote:** "Although not specified in the protocol, a worst-case imputation method was used for missing data to conform with the intention-to-treat analysis."<br>**Quote:** "1315 patients with suspected infections were screened for eligibility, of whom 630 were enrolled and randomly assigned to the procalcitonin group (n=311 patients) or the control group (n=319; figure 2). Four patients in the procalcitonin group and five in the control group were subsequently excluded from the analysis."<br>**Quote:** "Only two patients were lost to follow-up, one from each group. In the procalcitonin group, the patient died on day 25 but no information was available about antibiotic exposure for days 15–25. From worst-case imputation, this patient was judged to have received antibiotics until death. In the control group, the patient was lost to follow-up on day 22 but was judged to have survived until day 60, without receiving any antibiotics after day 22."<br>**Comment:** Just few exclusions/attrition; mortality unlikely to be affected by this.<br>Worst-case imputation method seems appropriate.
Selective Reporting (Reporting Bias)	Low risk	**Primary outcomes mentioned in protocol and mentioned in published article:**<br>-Exposition to antibiotics, defined by antibiotic-free days<br>-28-day mortality<br>-60-day mortality<br>**Secondary outcomes mentioned in protocol and mentioned in published article:**<br>-Consumption of antibiotics expressed as the Defined Daily Dose/1000 ICU-days<br>-The length of ICU and hospital stay<br>-The evolution of SOFA score parameters<br>-The number of mechanical ventilation-free days<br>-The percentage of emerging multiresistant bacteria between D1 and D28, as assessed by microbiologic examination of all clinical samples<br>-The percentages of relapses of infection<br>**Secondary outcomes mentioned in protocol and not mentioned in published article:**<br>-The acquisition cost of antibiotics<br>**Secondary outcomes mentioned in published article and not mentioned in protocol:**<br>-Percentage of patients with superinfection<br>-Duration of first episode of antibiotic treatment

		-Duration of antibiotic treatment according to infectionsite<br>**Quote:** “Although not prespecified in the protocol, we did several other exploratory subgroup analyses on the basis of age, sex, microbiologically documented infections, presence or absence of one or more positive blood cultures, septic shock, mechanical ventilation, and SOFA score at inclusion.“<br>**Comment:** These are no primary outcomes and just additional analysis.<br>**Quote:** “Last, no formal cost-effectiveness evaluation was done.”<br>**Comment:** Judgment "low risk" because “the acquisition cost of antibiotics” (protocol) is not part of my meta-analysis.<br>**Registration of study:** http://www.Clinicaltrials.gov: NCT00472667
Other Bias	Low risk	**Quote:** “The study sponsors did not participate in the study design, data collection, data analysis, data interpretation, or writing of the report.”<br>**Quote:** “C-EL has received lecture fees from Brahms, and Merck Sharp & Dohme-Chibret. BR has served as a consultant for AstraZeneca, Merck Sharp & Dohme-Chibret, and Lilly. JC has received consulting and lecture fees from Pfizer, Brahms, Wyeth, Johnson & Johnson, Nektar-Bayer, and Arpida. MW has received consulting and lectures fees from Merck Sharp & Dohme-Chibret, Janssen-Cilag, Gilead, and AstraZeneca. All other authors declare that they have no conflicts of interest.”<br>**Comment:** No extreme baseline imbalance between groups.

Study: Hochreiter et al. 2009

Bias	Authors´ Judgment	Support for Judgment
Random Sequence Generation (Selection Bias)	Unclear risk	**Quote:** “Patients were randomly assigned to either a PCT-guided (study group) or a standard (control group) antibiotic regimen.”<br>**Quote:** "Die Patienten wurden per Losverfahren entweder der PCT-gesteuerten Gruppe oder der Kontrollgruppe zugeteilt."
Allocation Concealment (Selection Bias)	Unclear risk	**Comment:** No information given.
Blinding of Participants and Personnel (Performance Bias)	Low risk	**Comment:** Patients treated according to a strict protocol.<br>**Quote:** “Antibiotic therapy in the PCT-guided group was discontinued if clinical signs and symptoms of infection improved and PCT decreased to less than 1 ng/ml, or if the PCT value was more than 1 ng/ml, but had dropped to 25 to 35% of the initial value over three days. In the control group, antibiotic treatment was applied as standard regimen over eight days. Irrespective of the study group and at any time point, the physician in charge had the option to proceed with or adjust the antibiotic treatment, if there were clinical reasons to do so.”
Blinding of Outcome Assessment (Detection Bias)	Unclear risk	**Comment:** No information given.
Incomplete Outcome Data (Attrition Bias)	Low risk	**Quote:** “Of 395 patients screened, a total of 110 patients fulfilling the inclusion criteria were entered in the study from January 2006 to March 2007.”<br>**Quote:** "Fifty-seven patients were randomly assigned to the PCT-guided group and 53 to the control group.”<br>**Comment:** No exclusion and attrition after inclusion of patients mentioned. No missing outcome data of all 110 included patients.
Selective Reporting (Reporting Bias)	Low risk	**Primary outcome was reported according to the study protocol:**<br>-Duration of antibiotic therapy<br>**Outcomes mentioned in published article and not mentioned in protocol:**<br>-Length of intensive care treatment<br>**Registration of study:** http://www.isrctn.org: ISRCTN10288268

Other Bias	Low risk	**Quote:** "In the present prospective, randomised open study, both treatment groups were comparable in terms of age, gender distribution, diagnoses, disease severity as reflected by SAPS II, and outcome (Table 1). The distribution of antibiotic classes used was comparable as well (Table 2)."<br>**Comment:** No extreme baseline imbalance between groups.<br>**Quote:** "SS has served as consultant and has received payments from BRAHMS AG for speaking engagements. All other authors declare no conflicts of interest."

Study: Jensen et al. 2011

Bias	Authors´ Judgment	Support for Judgment
Random Sequence Generation (Selection Bias)	Low risk	**Quote:** “Randomization was performed 1:1 using a computerized algorithm created by the database manager with concealed block size, prestratified for site of recruitment, initial Acute Physiology and Chronic Health Evaluation, and age (entered in an encrypted screening form in a passwordprotected Web site)”
Allocation Concealment (Selection Bias)	Low risk	**Quote:** “Randomization was performed 1:1 using a computerized algorithm created by the database manager with concealed block size, prestratified for site of recruitment, initial Acute Physiology and Chronic Health Evaluation, and age (entered in an encrypted screening form in a passwordprotected Web site); investigators were masked to assignment before randomization.”
Blinding of Participants and Personnel (Performance Bias)	Low risk	**Comment:** Patients treated according to a strict protocol.<br>**Quote:** “Patients were randomized either to the “standard-of-care-only arm,” receiving treatment according to the current international guidelines and blinded to procalcitonin levels, or to the “procalcitonin arm,” in which current guidelines were supplemented with a drug-escalation algorithm and intensified diagnostics based on daily procalcitonin measurements.”<br>**Quote:** “The interventional algorithm was available at all sites and all investigators were trained in it. Additionally, everyday, all sites were contacted by telephone (365 days/yr) to assure that interventions were conducted according to the algorithm.”<br>**Quote:** “The main principle in the intervention algorithm was whenever an “alert procalcitonin” occurred, 1) to substantially increase the antimicrobial spectrum covered and 2) to intensify the diagnostic effort to find uncontrolled sources of infection, in this way interpreting an “alert procalcitonin” as a warning of uncontrolled infection. “Alert procalcitonin” was defined as a procalcitonin >1.0 ng/mL that was not decreasing at least 10% from the previous day. At baseline, a single procalcitonin measurement of >1.0 ng/mL was considered to be “alert procalcitonin.” Both arms received antimicrobial therapy according to current guidelines.”<br>**Quote:** “In the procalcitonin group, 256 of 312 (82.1%) of patients with baseline “alert procalcitonin” received antimicrobials according to the available procalcitonin measurement and the intervention algorithm“<br>**Quote:** „Of patients in the standard-of-care-only arm, who were judged to have severe sepsis or septic shock at baseline, 172 of 209 (82.4%) received antimicrobials according to empiric “standard-of-care” principles”

Blinding of Outcome Assessment (Detection Bias)	Low risk	**Quote:** "Investigators, treating physicians and the coordinator were unaware of outcomes during the study as were all procalcitonin measurements in the standard-of-care- only (control) group."
Incomplete Outcome Data (Attrition Bias)	Low risk	**Quote:** "Good Clinical Practice was applied. As part of this, double-keying, monitoring, and correction of errors and missing data were done in collaboration between the investigator and a clinical monitor."<br>**Quote:** "The primary analysis includes all patients who were randomized."<br>**Quote:** "Follow-up for the primary end point was complete (100.0%) for all patients randomized (604 in the procalcitonin group, 596 in the standard-of-care-only group)."<br>**Comment:** Regarding mortality no missing outcomes/exclusions/attrition.
Selective Reporting (Reporting Bias)	High risk	**Primary outcome was reported according to the study protocol:**<br>-28-day mortality<br>**Secondary outcomes mentioned in protocol and mentioned in published article:**<br>-60-day mortality<br>-Consumption of antimicrobial chemotherapy<br>-Prevalence of complications to infection: 2) severe sepsis, 3) septic shock: data only for both outcomes together available<br>**Secondary outcomes mentioned in protocol and not mentioned in published article:**<br>-90 day mortality<br>-120 day mortality<br>-180 day mortality<br>-Prevalence of complications to infection: 1)sepsis, 2)severe sepsis, 3) septic shock, 4)MODS, 5)DIC: data for severe sepsis/septic shock only in combination for both groups available.<br>-Use of diagnostic imaging during admission to the ICU<br>-Quality of life post-ICU<br>**Quote:** "Patients in the procalcitonin group were more likely to have additional cultures performed within 24 hrs after an "alert procalcitonin" than patients in the standard-of-care-only group: 81.5% vs. 66.7% ($p < .001$) but no more likely to have imaging studies or surgical interventions (data not shown)."<br>**Secondary outcomes mentioned in published article and not mentioned in protocol:**<br>-Median intensive care unit admission length<br>-Mean time to appropriate antimicrobials<br>-Need for organ support (mechanical ventilation, vasopressors/inotropics, glomerular filtration rate <60 mL/1.73 m², dialysis)

		-Other organ failure measures (bilirubin>1.2 mg/dL, Glasgow Coma Score<13)<br>-Rate of infection at the time of discharge or at day 28<br>-"alert procalcitonin" at baseline as predictor of 28-day all-cause mortality<br>-Infection/host response by clinical assessment<br>**Quote:** "the sensitivity of the procalcitonin test for infection estimated in this trial was as low as 59%"<br>**Quote:** "In the present trial, the procalcitonin strategy increased costs substantially"<br>**Registration of study:** http://Clinicaltrials.gov: NCT00271752
Other Bias	Low risk	**Comment:** No extreme baseline imbalance between groups.<br>**Quote:** "Dr. Jensen received speaker fee and travel reimbursement from Brahms Diagnostica and received an unrestricted grant for the organization for sample transport and analysis. The remaining authors have not disclosed any potential conflicts of interest."

Study: Nobre et al. 2008

Bias	Authors´ Judgment	Support for Judgment
Random Sequence Generation (Selection Bias)	Low risk	**Quote:** "The randomization was performed using a computer-based random number generation."
Allocation Concealment (Selection Bias)	Low risk	**Quote:** "Allocation was issued using opaque, sealed, numbered envelopes."
Blinding of Participants and Personnel (Performance Bias)	Unclear risk	**Quote:** "We conducted a randomized, controlled, open interventional trial"<br>**Comment:** Patients treated according to a strict protocol.<br>**Quote:** "All patients received initial antibiotic therapy based on local guidelines and susceptibility patterns, according to the decision of the treating physician, who was unaware of the patient's initial PCT levels."<br>**Quote:** "In patients randomly assigned to the intervention group, antibiotics were stopped when PCT levels had decreased 90% or more from the initial value (if clinicians agreed) but not before Day 3 (if baseline PCT levels were >1 mg/L) or Day 5 (if baseline PCT levels were >1 mg/L). In control patients, clinicians decided on the duration of antibiotic therapy based on empirical rules."<br>**Quote:** "Of note, the final decision concerning the antibiotic therapy duration was always left to the discretion of the physician in charge."<br>**Quote:** " "Algorithm overruling" ' in the PCT group (i.e., treating physician refused to stop the antibiotics, although the stopping rules allowed this) occurred in 6 of 31 (19%) patients of the PCT group."<br>**Quote:** "It is worthy to stress that in 19% of patients allocated to the PCT group, treating physicians refused to stop the antibiotics, although the stopping rules allowed this (Table E4). We can consider protocol overruling (i.e., prolongation of the antibiotic therapy by the treating physician beyond the stopping rule) as a ''conservative bias.''"<br>**Quote:** "In no case assigned to the PCT group did the treating physicians stop antibiotics before the time when patients had reached the criteria for discontinuation based on the algorithm."
Blinding of Outcome Assessment (Detection Bias)	Unclear risk	**Comment:** No information given.
Incomplete Outcome Data (Attrition Bias)	Low risk	**Quote:** "Seventy-nine of the 282 patients screened for eligibility were randomized; 39 in the PCT group and 40 in the control group (Figure 1)."<br>**Quote:** "Primary endpoints were first analyzed on the basis of an intention-to-treat analysis, including

		all randomized patients."<br>**Quote**: "Because of dropouts (early deaths and newly discovered complicated infections), 68 patients (control group, n=37, and PCT group, n=31) reached a time when a decision to stop antibiotics could be taken (PCT group) or potentially be taken (control group) based on the relative decrease of daily measured PCT levels (per-protocol analysis, Figure 1)."<br>**Quote:** "the number of dropouts observed in this trial was imbalanced between the two groups (8 patients in the PCT group vs. 3 patients in the control group, P=0.197)."<br>**Comment:** In the meta-analysis we consider only the intention-to-treat-analysis. Therefore there are no missing data. However, it remains unclear how dropouts have been included in the intention-to-treat-analysis.
Selective Reporting (Reporting Bias)	Unclear risk	**Primary outcome was reported according to the study protocol:**<br>-Duration of antibiotic treatment<br>-Total antibiotic exposure<br>**Primary outcomes not mentioned in protocol and mentioned in published article:**<br>-Days alive without antibiotics<br>**Secondary outcomes mentioned in protocol and mentioned in published article:**<br>-Clinical cure<br>-28-day mortality<br>-Length of hospital stay<br>-Rate of nosocomial super-infection<br>**Secondary outcomes mentioned in protocol and not mentioned in published article:**<br>-Costs of antimicrobial therapy (in CHF)<br>-Isolation of multi-resitant microorganisms (in clinical isolates per 100 patient-days)<br>**Secondary outcomes mentioned in published article and not mentioned in protocol:**<br>-In-hospital mortality<br>-Sepsis-related death<br>-Primary infection relapse rate<br>-Length of ICU stay<br>**Registration of study:** http://Clinicaltrials.gov: NCT00250666
Other Bias	Low risk	**Quote:** "The reasons for exclusion were as follows: (1) microbiologically documented infections caused by Pseudomonas aeruginosa, Acinetobacter baumanni, Listeria spp., Legionella pneumophila, Pneumocystis jiroveci, or Mycobacterium tuberculosis, for which a prolonged duration of antibiotic therapy is standard-of-care (17); […] or (8) absence of antimicrobial treatment despite clinical

		suspicion of sepsis.”<br>**Quote:** “For security, we excluded difficult-to-treat microorganisms, infections that are known to require prolonged antibiotic therapy, and severely immunocompromised and neutropenic patients.”<br>**Comment:** No extreme baseline imbalance between groups.<br>**Quote:** “V.N. does not have a financial relationship with a commercial entity that has an interest in the subject of this manuscript. S.H. and J.P. received a research grant from BRAHMS AG ($50,000). BRAHMS AG had no influence on study design, data analysis, or final preparation of this manuscript. S.H. received speaker honoraria ($1,500) from BRAHMS AG. J.-D.G. does not have a financial relationship with a commerical entity that has an interest in the subject of this manuscript. P.R. does not have a financial relationship with a commercial entity that has an interest in the subject of this manuscript. J.P. received speaking honoraria from BRAHMS AG (less than $1,000 in 2006 and 2007).”

Study: Schroeder et al. 2009

Bias	Authors´ Judgment	Support for Judgment
Random Sequence Generation (Selection Bias)	Unclear risk	**Quote:** "Thereafter, patients were randomly assigned to either PCT-guided antibiotic treatment or a control group receiving standard antibiotic therapy."
Allocation Concealment (Selection Bias)	Unclear risk	**Comment:** No information given.
Blinding of Participants and Personnel (Performance Bias)	Low risk	**Comment:** Patients treated according to a strict protocol.<br>**Quote:** "For either group, a calculated antibiotic regimen according to the underlying infectious pathology was applied."<br>**Quote:** "In the PCT-guided group, antibiotic therapy was discontinued if clinical signs and symptoms of sepsis improved and PCT values either had decreased to 1 ng/ml or less or had dropped to 25–35% of the initial PCT concentration over three consecutive days. In the control group, antibiotic treatment was discontinued according to clinical signs and empiric rules. Independent from the study protocol, the physician in charge was always free to decide to continue or change the antibiotic regimen upon clinical judgement."
Blinding of Outcome Assessment (Detection Bias)	Unclear risk	**Comment:** No information given.
Incomplete Outcome Data (Attrition Bias)	Low risk	**Quote:** "27 of 125 screened patients met the inclusion criteria"<br>**Quote:** "Finally, 14 patients were randomly assigned to the PCT-guided treatment group and 13 patients to the control group."<br>**Comment:** No missing outcome data.
Selective Reporting (Reporting Bias)	Unclear risk	**Outcomes reported in the methods section and in the results section of the article:**<br>-Length of ICU stay<br>-Duration of antibiotic treatment<br>-Hospital mortality<br>-SOFA<br>-SAPS II<br>**Outcomes reported in the methods section but not in the results section of the article:**<br>-Length of hospital stay

		**Outcomes reported in the results section but not in the methods section of the article:**<br>-Cost of antibiotic treatment<br>**Comment:** No study protocol published.<br>Trial not registered at http://www.clinicaltrials.gov or at http://www.isrctn.org
Other Bias	Low risk	**Comment:** No extreme baseline imbalance between groups.<br>**Quote:** “The corresponding author declares speaking engagements for BRAHMS AG. All other authors declare no conflict of interest.”

Study: Svoboda et al. 2007

Bias	Authors´ Judgment	Support for Judgment
Random Sequence Generation (Selection Bias)	Low risk	**Quote:** "Patients were randomly assigned to study groups by means of a computer generated random number table to generate a random treatment list."
Allocation Concealment (Selection Bias)	Low risk	**Quote:** "Treatment regimens were included in opaque sealed numbered envelopes and envelope with the lowest number was always used for consecutive patients."
Blinding of Participants and Personnel (Performance Bias)	Low risk	**Comment:** Patients treated according to a strict protocol.<br>**Quote:** "Of 453 screened patients, 72 patients fulfilled the inclusion criteria and were randomized into 2 study groups: in the first group (PCT, n=38), more important role in the treatment decision was given to PCT level (severe sepsis with PCT >2ng/mL signalized bacteremia and pushed us to change antibiotics and catheters; severe sepsis with PCT <2ng/mL prompted to ultrasonography and/or CT, followed by repeated surgical treatment – drainage, re-operation - if localized infection was confirmed). The control group (CON, n=34) was treated by standard evaluation of all parameters by consultant surgeon according to contemporary treatment protocol of our institute."
Blinding of Outcome Assessment (Detection Bias)	Unclear risk	**Comment:** No information given.
Incomplete Outcome Data (Attrition Bias)	Low risk	**Quote:** "Of 453 screened patients, 72 patients fulfilled the inclusion criteria and were randomized into 2 study groups"<br>**Quote:** "Seventy-two patients with severe sepsis after abdominal surgery or after surgery for multiple trauma were randomly allocated in group PCT (n=38) or CON (n=34)."<br>**Comment:** no missing outcome data.
Selective Reporting (Reporting Bias)	Low risk	**Same outcomes reported in the methods section and the results section of the article:**<br>-28-day mortality<br>-Duration of stay in the ICU<br>-Days of mechanical ventilation<br>-SOFA score<br>**Comment:** No study protocol published.<br>Trial not registered at http://www.clinicaltrials.gov or at htpp://www.isrctn.org

		Trial registered at http://www.sukl.eu
Other Bias	Low risk	**Comment:** No extreme baseline imbalance between groups.<br>**Quote:** “The study was supported by Grant of IGA MZ CR ND 7676-3.”
