# Supplementary figures and images for: Procalcitonin-guided therapy in intensive care unit patients with severe sepsis and septic shock – a systematic review and meta-analysis
Source: Crit Care. 2013 Dec 11;17(6):R291. doi: 10.1186/cc13157 (PMC4056085; doi:10.1186/cc13157)

Additional File 2. Risk of Bias Graph

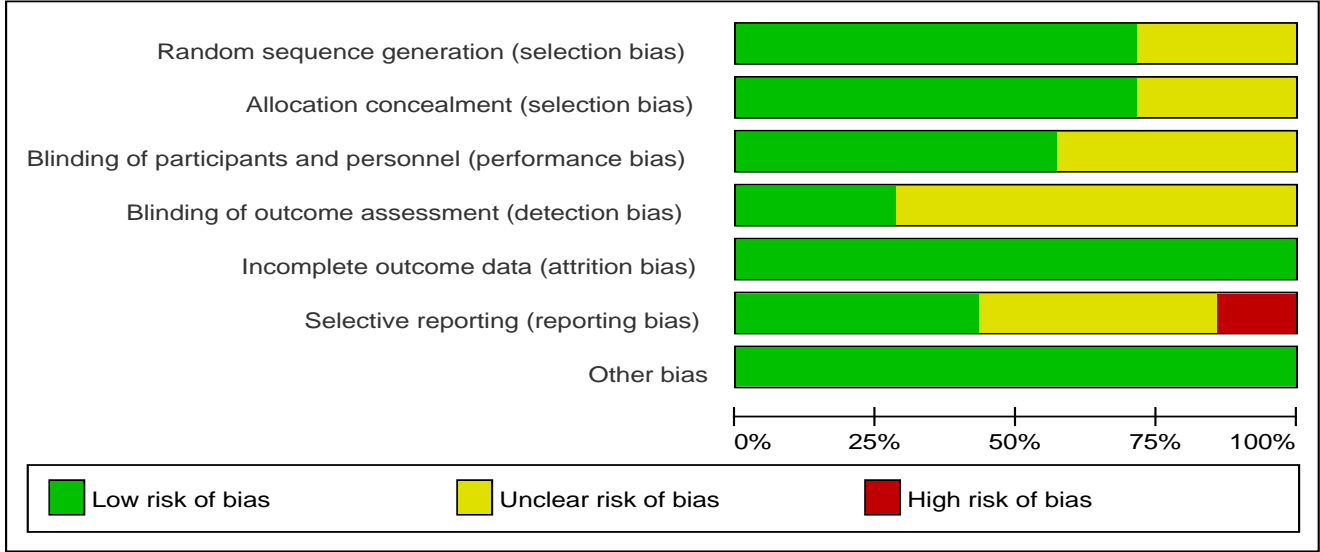

Supplement: Additional file 2 — Risk of bias graph. Cochrane Collaboration tool for assessing risk of bias. Review authors’ judgments about each risk of bias item presented as percentages across all included studies. [file cc13157-S2.pdf]
